# Supplementary material for: Trait-based community assembly and succession of the infant gut microbiome
Source: Nat Commun. 2019 Feb 1;10:512. doi: 10.1038/s41467-019-08377-w (PMC6358638; doi:10.1038/s41467-019-08377-w)
Supplement: Supplementary file 1 — Supplementary Information [file 41467_2019_8377_MOESM1_ESM.pdf]

Supplementary Tables and Figures for “Trait-based community assembly and succession of the infant gut microbiome” by Guittar et al.

Trait	IC x SBT	SBT x IC	SCP x IC
16S gene copy number	0.94	0.94	0.95
B vitamin number	0.94	0.94	0.87
GC content	0.98	0.98	0.98
Gene number	0.97	0.97	0.97
Genome size	0.95	0.95	0.97
Gram-positive	0.98	0.98	0.98
Length	0.96	0.96	0.92
Motility	0.96	0.96	0.93
Oxygen tolerance	0.97	0.97	0.98
Sporulation score	0.97	0.97	0.94
Temperature optimum	0.92	0.92	0.94
Width	0.94	0.94	0.90

Supplementary Table 1 | Comparisons of trait prediction algorithms: Pearson correlations coefficients among trait value estimates arising from three methods of hidden state character prediction. Predictions were made for all OTUs in gut microbiome samples and/or from the Living Tree Project which could not be associated with directly observed trait data based on their Latin binomials. Only predictions falling within acceptable phylogenetic distances to taxa with directly observed trait data were considered (refer to Methods), thus sample size ranged from N = 8122 (Oxygen tolerance) to N = 13511 (B vitamin number). Traits unamenable to hidden state prediction were omitted. IC: Independent Contrasts; SBT: Subtree Averaging; SCP: Weighted Squared-change Parsimony.

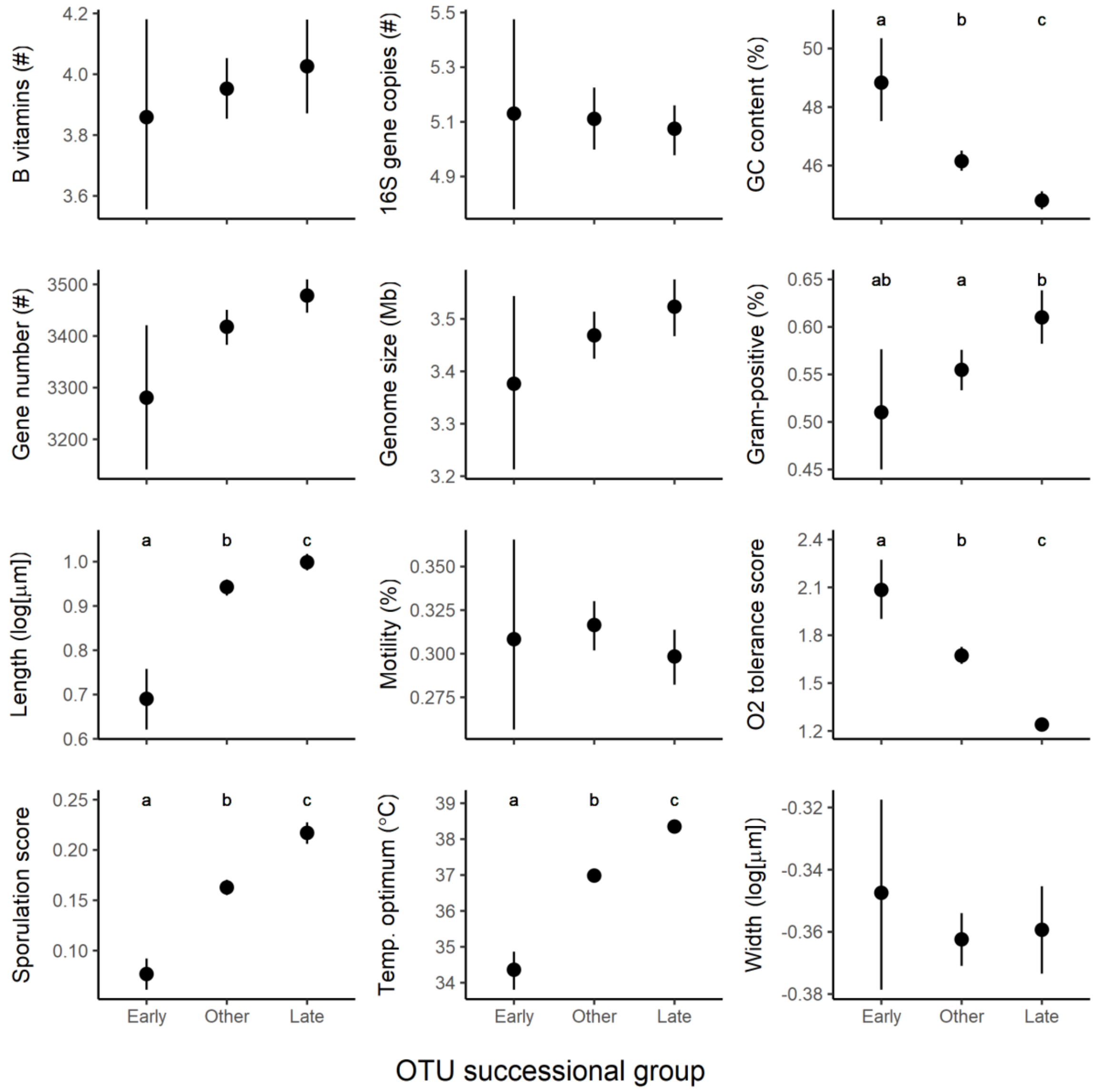

Supplementary Figure 1 | Early and late successional specialists differ in traits: Mean trait values of OTUs from early (N = 139), middle or no-trend (i.e., Other; N = 209), and late (N = 393) successional stages. Unlike elsewhere in this study, calculations are not weighted by relative abundances. OTUs were assigned to successional groups according to whether they exhibited significant positive or negative trends in overall abundance over succession based on linear models. Vertical bars show 95 percent confidence intervals. Letters (a/b/c) denote significance ($p < 0.05$) between successional categories for each trait based on Welch t-tests.

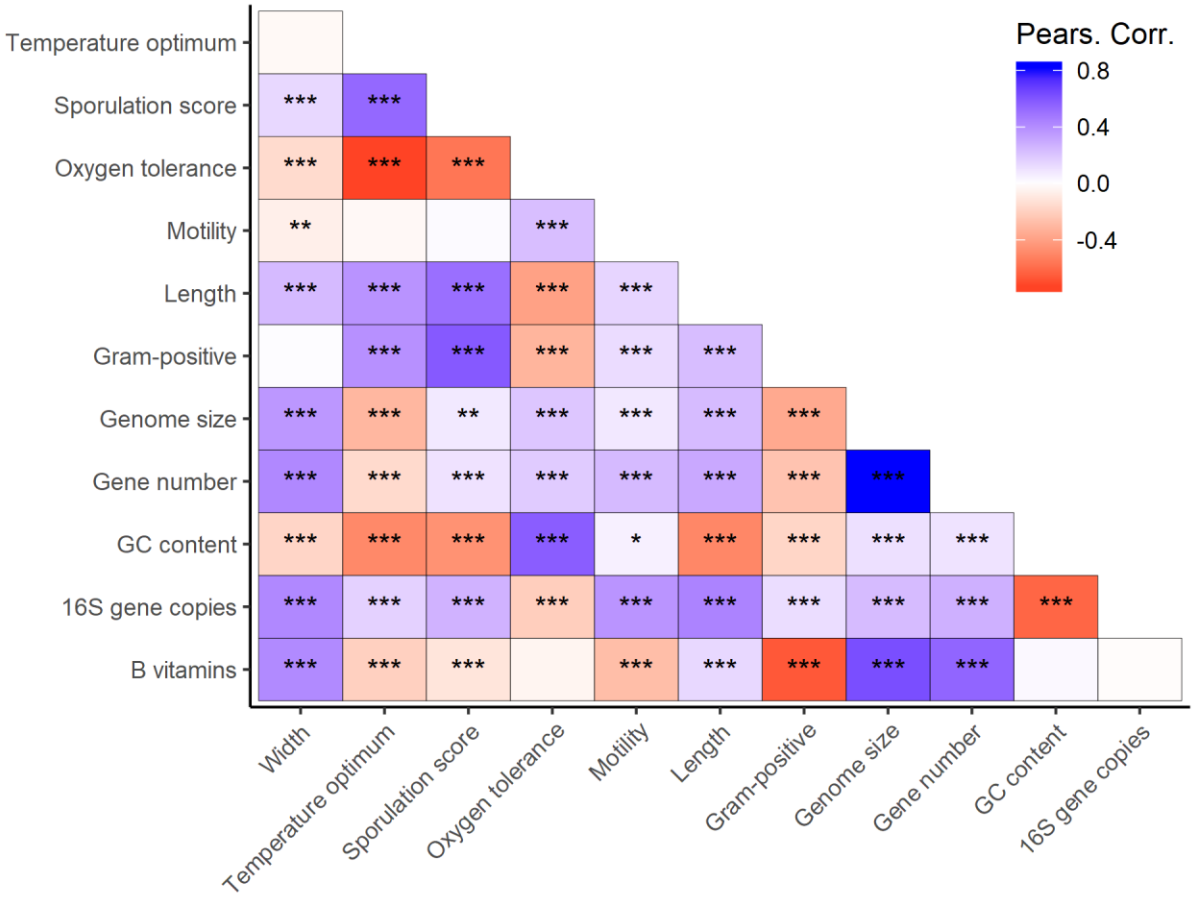

Supplementary Figure 2 | Pearson trait correlations among taxa in this study: Only trait predictions made within the maximum allowable phylogenetic distance were used (Table 2). The number of comparisons for each trait pair varied depending on data availability and ranged from N = 979 (16S rRNA gene copy number and B vitamin number) and N = 2298 (length and GC content). Asterisks denote significance (*: adjusted $p < 0.05$; **: adjusted $p < 0.01$; ***: adjusted $p < 0.001$).

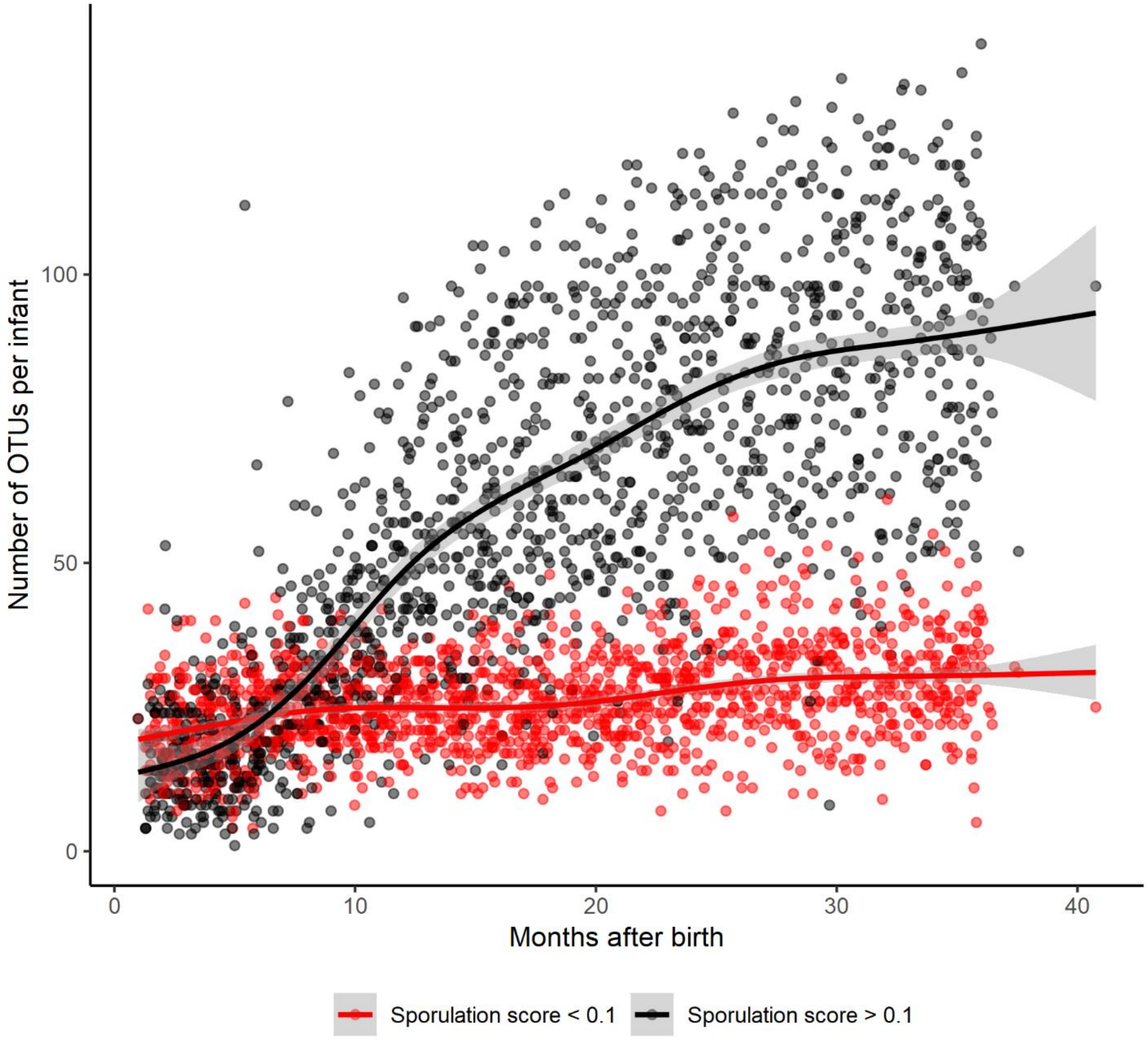

Supplementary Figure 3 | Numbers of sporulating taxa increase over succession. Each point reflects the number of taxa with sporulation scores over or under 0.1 in a given sample from a given infant. Smooth trendlines were fitted using generalized additive models. The rise in the number of taxa with sporulation scores > 0.1 over time illustrates that it is not only an increase in the abundance of preexisting taxa driving the observed increase in abundance-weighted sporulation score (Fig. 2), but also an increase in taxonomic richness.

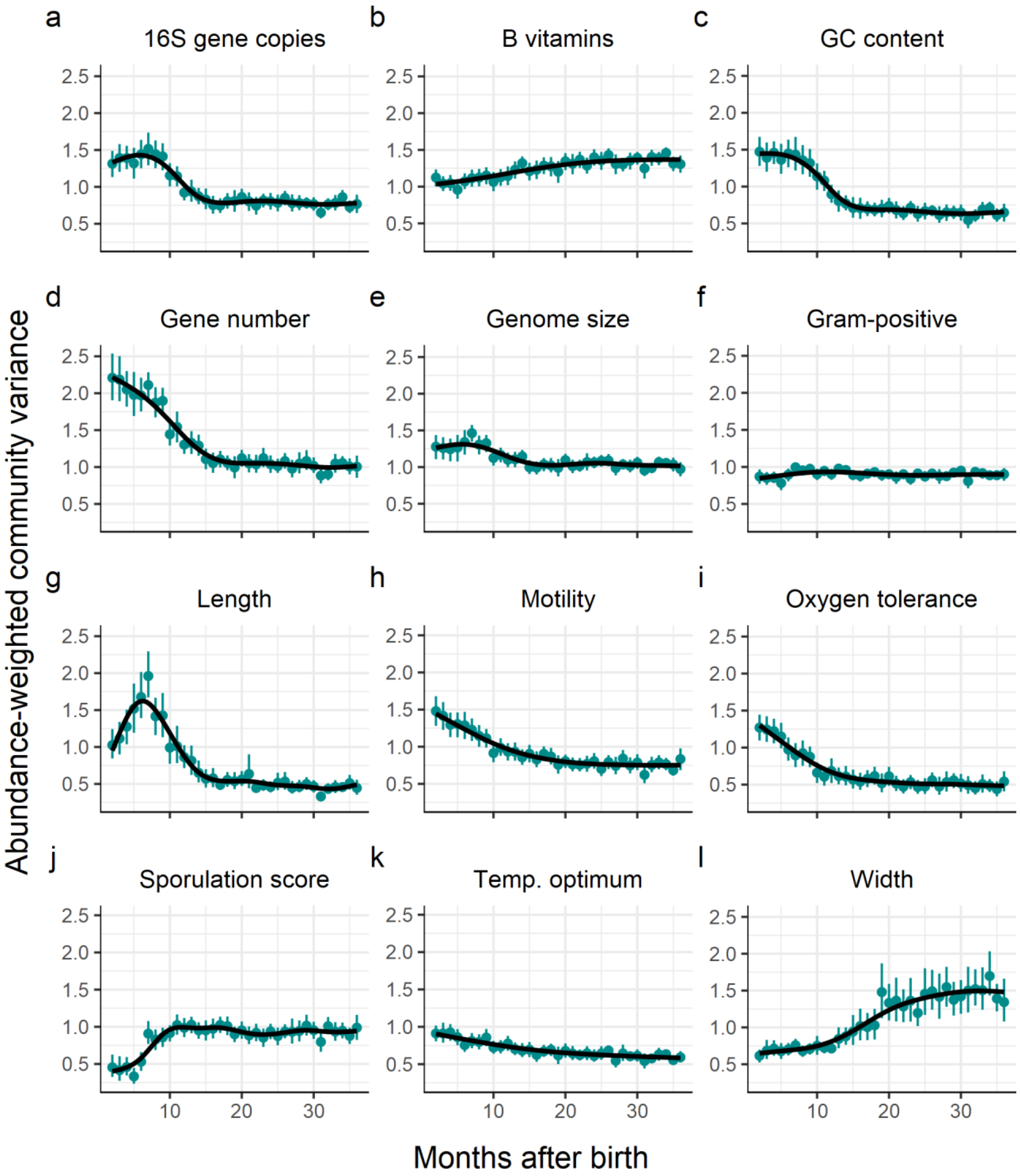

Supplementary Figure 4 | Patterns of community trait variance over succession. Variance is calculated on the predicted trait values of the cells putatively present in a community sample; i.e., the trait values of the taxa from a given sample, weighted by their relative abundances in that sample. To enable across-trait comparisons, sets of predicted values for each trait are scaled by the standard deviation of trait

values observed in the taxa from this study. Circles show average variance of samples in that month. The number of data points in a month varies depending on the number of samples collected, ranging from N = 27 to N = 59. Vertical lines show 95 percent confidence intervals. Trendlines show shifts in community trait variance over time, calculated using generalized additive models.

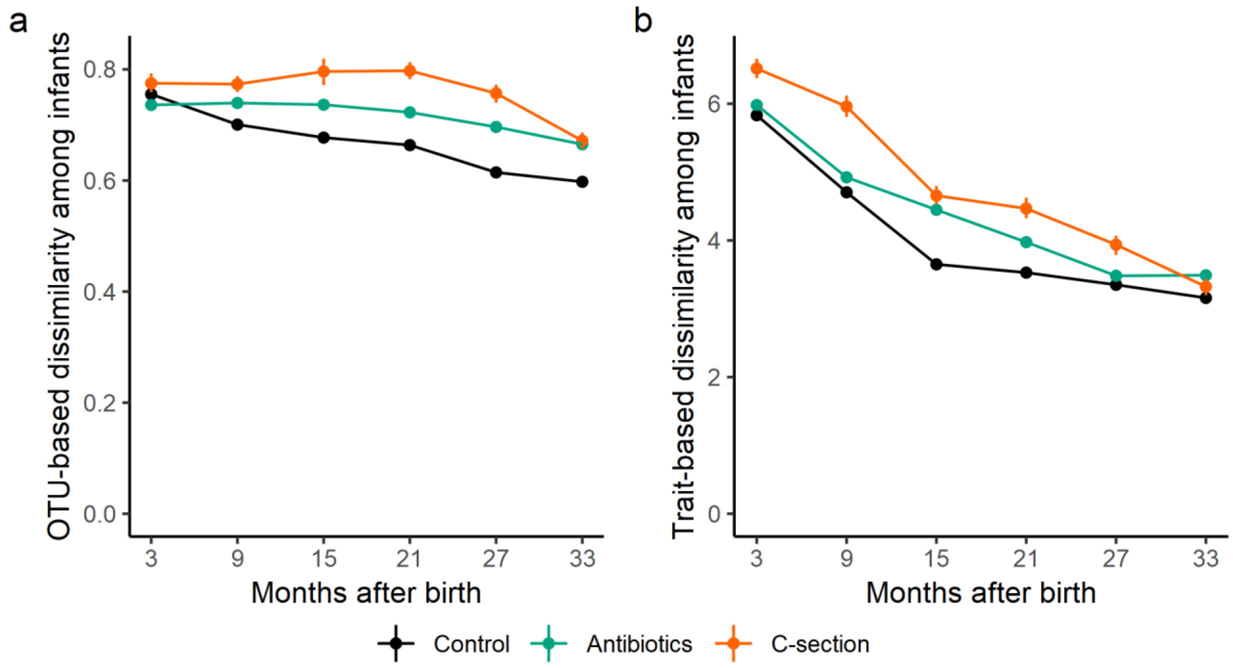

Supplementary Figure 5 | Microbiomes vary more among C-section and antibiotic-treated infants. Mean dissimilarities in gut community compositions were calculated for all sample pairs within six-month periods within treatment groups, excluding pairs of samples from the same infants. OTU-based differences were calculated using Bray-Curtis dissimilarity. Trait-based dissimilarities were calculated using multidimensional Euclidean distance after scaling each trait so it contributes equally. N equals the number of all possible sample pairs within each treatment; within each six-month period, there were between 25 to 31 total samples from six infants delivered by C-section, 66 to 91 total samples from 18 infants that received at least 50 days of antibiotic treatment, and 72 to 93 total samples from 18 control infants that were delivered vaginally and did not receive antibiotics. Due to the large number of pairwise comparisons, and therefore sample sizes, both treatment groups are almost always significantly different from the control infant group during all time periods.

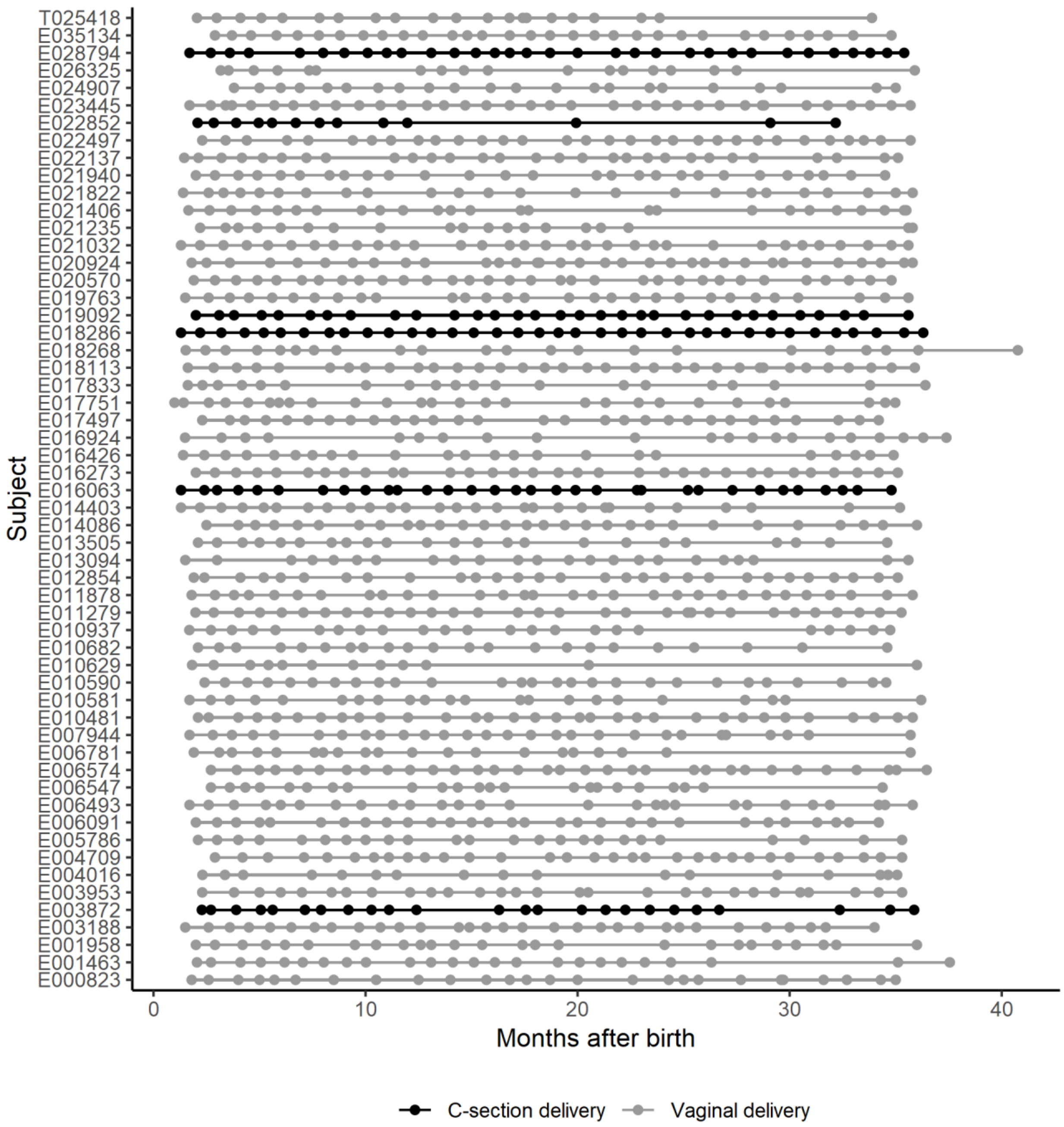

Supplementary Figure 6 | Sampling times and delivery modes of infants in this study. Each horizontal line is a subject; each filled circle is a sample.

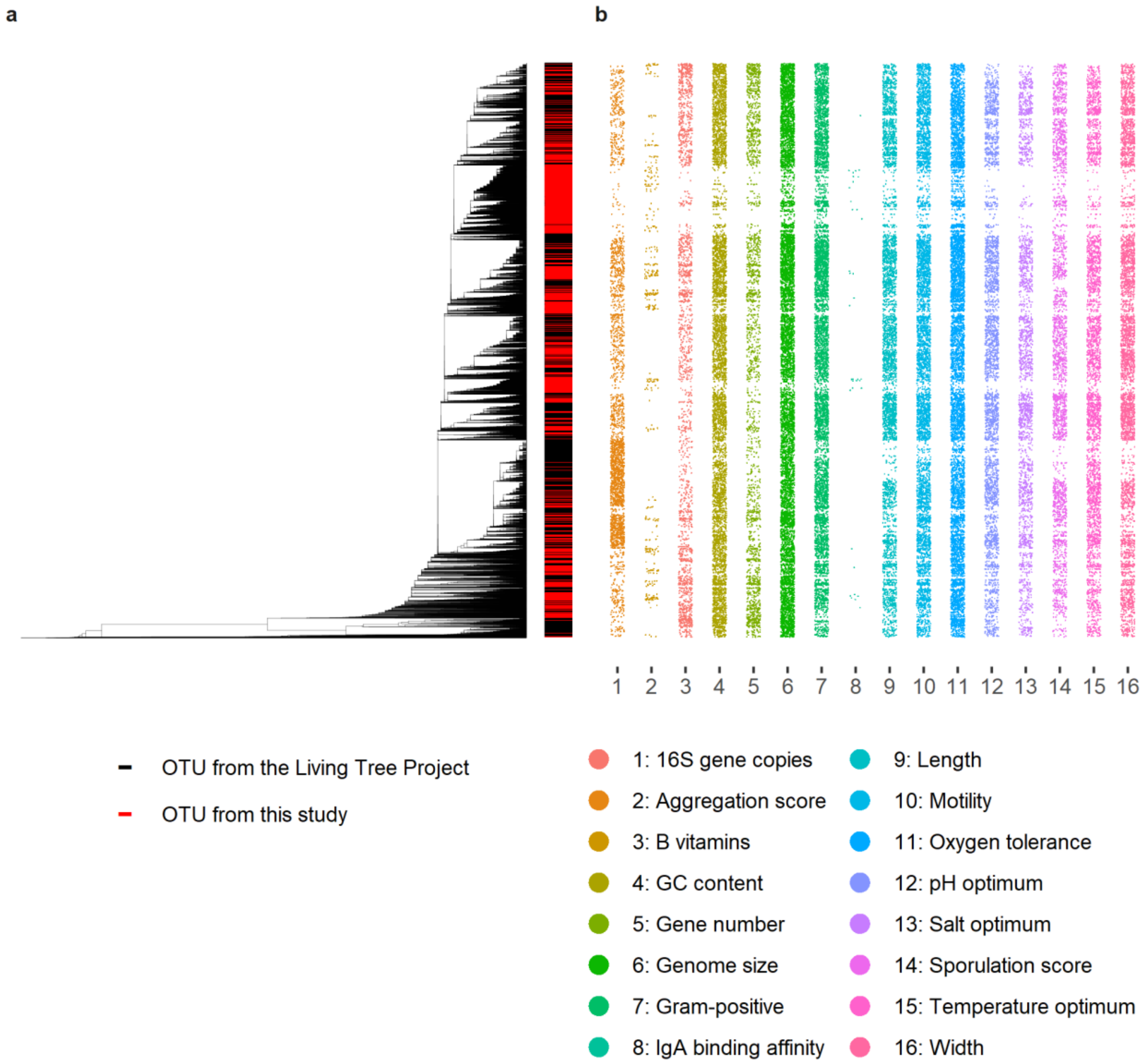

Supplementary Figure 7 | Phylogenetic coverage of trait observations. (a) A phylogenetic tree constructed from a distance matrix of sequence similarity in the 16S rRNA V4 region. The tips of the tree comprise the 3311 OTUs in this study (before any were removed during the rarefaction process) and the 13900 OTUs from the 132 release of the Living Tree Project (Muñoz et al. 2011). The color of the horizontal lines to the right of the tips of the tree reflects the OTU source. **(b)** A jittered scatterplot illustrating the number and phylogenetic extent of individual trait observations compiled from databases and the literature; refer to Table 1 for more details.

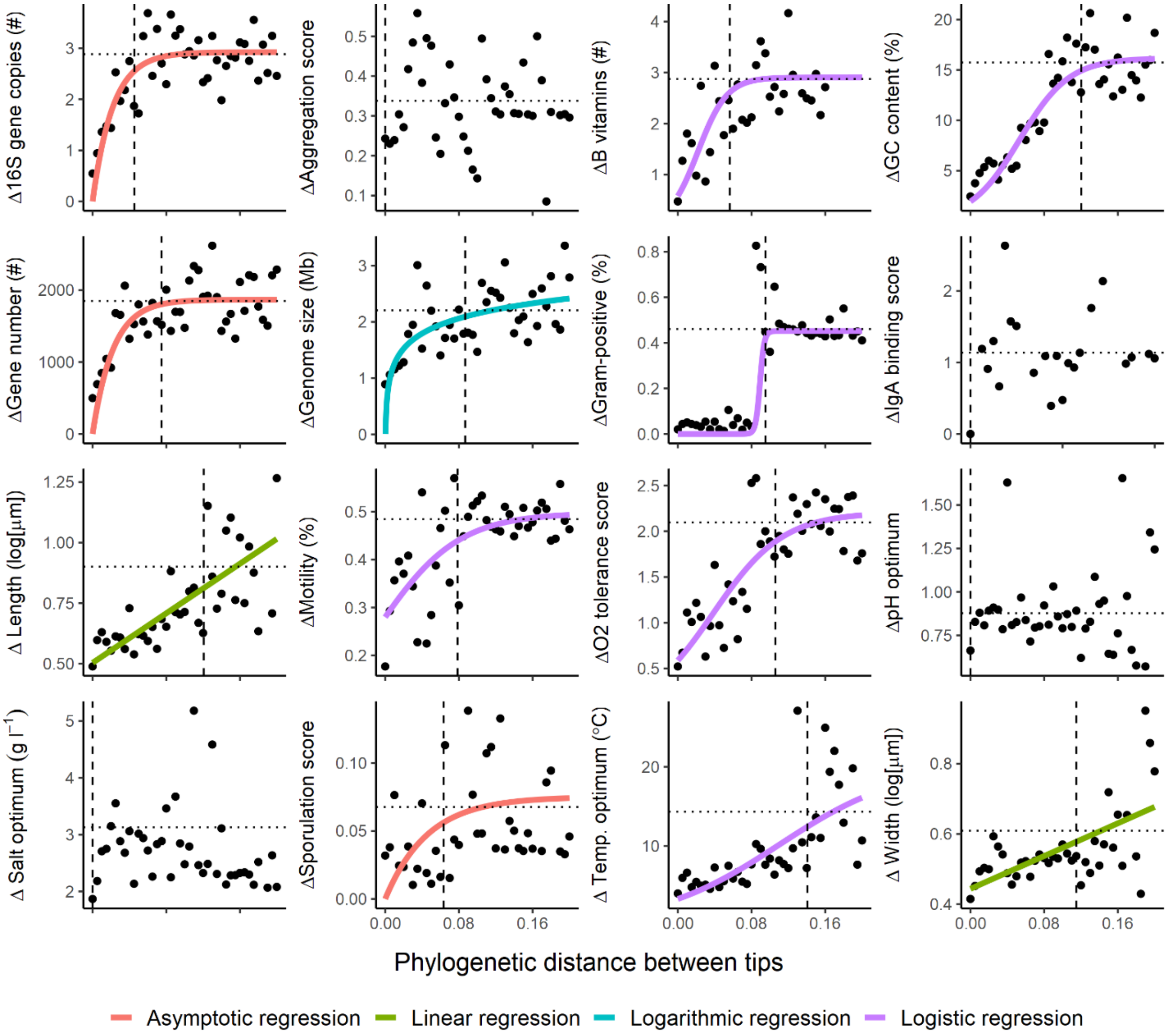

Supplementary Figure 8 | Determining the limits of phylogenetic inference for each trait. Each filled black circle shows the mean trait-based difference among 1000 randomly selected pairs of taxa that fall within 0.5 percent bins of increasing sequence dissimilarity in the 16S rRNA V4 region. Horizontal dotted lines show null expectations for each trait, equal to the mean trait-based difference across all pairs of taxa with at least 10 percent sequence dissimilarity. The size (i.e., the number of tips) of the phylogeny used for each trait varied, and was equal to the number of OTUs with known trait values. Hence, the number of pairwise comparisons differed by trait, ranging from 666 (IgA binding affinity) to 463621 (GC content). Colored trendlines show the best-fit models for each trait, as determined by AIC, selected from

a suite of four basic models: (1) null model, (2) linear regression, (3) logarithmic regression, (4) asymptotic regression, constrained to pass through the origin, and (5) logistic regression. Refer to Methods section for model equations. Vertical dashed lines mark the maximum phylogenetic distances at which unknown trait values were inferred for each trait in this study, and were calculated as the point at which the best-fit model reached 90 percent of the null expectation of pairwise trait dissimilarity.

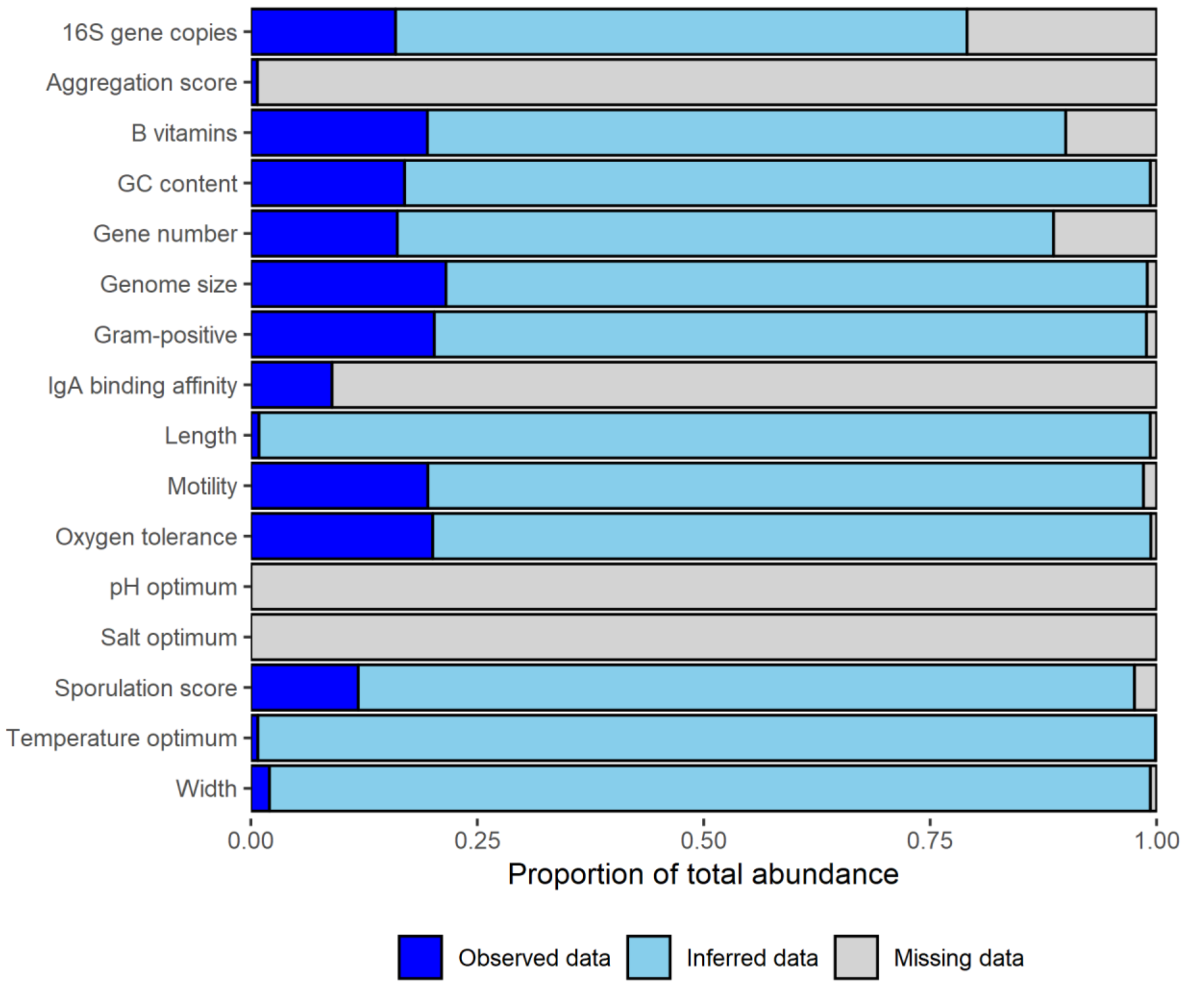

Supplementary Figure 9 | Proportions of sequences with observed or inferred trait data. Proportions of sequences in this study with trait data that were drawn from the literature (i.e., observed data), inferred using hidden state prediction (i.e., inferred data; see Methods), or were not inferred because the trait in question did not exhibit sufficient phylogenetic signal (Table 2, Supplementary Figure 8) and/or the taxa lacked close relatives with trait data (Supplementary Figure 7) and were therefore dropped from analyses (i.e., missing data).
