# Supplementary material for: Trait-based community assembly and succession of the infant gut microbiome
Source: Nat Commun. 2019 Feb 1;10:512. doi: 10.1038/s41467-019-08377-w (PMC6358638; doi:10.1038/s41467-019-08377-w)
Supplement: Supplementary file 3 — Description of Additional Supplementary Files [file 41467_2019_8377_MOESM3_ESM.pdf]

Description of Additional Supplementary Files

File name: Supplementary Data 1
Description: **Relative abundances of OTUs in infant microbiome samples.** Raw 16S rRNA V4 amplicon sequencing data from infant gut microbiome samples were processed using a USEARCH pipeline and clustered into operational taxonomic units (OTUs) at 97 percent similarity. Samples with fewer than 5000 initial sequences were excluded, and the remaining samples were rarefied to 5000 sequences, resulting in a drop from 3311 to 2416 OTUs. The 'subject' column can be used with Supplementary Data 2 to find associated subject metadata (e.g., delivery method, antibiotic treatment history), and the 't' column refers to the month of sampling. Available at https://doi.org/10.6084/m9.figshare.7499498.

File name: Supplementary Data 2
Description: **Infant metadata.** Information for each infant subject includes (1) the approximate total number of days of antibiotic treatment, assuming the duration of any given antibiotic treatment was seven days unless otherwise noted, (2) the country of origin, (3) the mode of delivery, (4) the treatment group assignment used in this study, and (5) references to the prior studies from which the metadata were drawn. Available at https://doi.org/10.6084/m9.figshare.7499525. Additional subject metadata is available in Kostic et al. 2016 (ref. 27 in the main text) and Yassour et al. 2016 (ref. 28 in the main text).

File name: Supplementary Data 3
Description: **SILVA-based taxonomic identities of infant microbiome OTUs.** Representative sequences of operational taxonomic units (OTUs) from infant microbiome samples were mapped to the SILVA v123 database using USEARCH version 10.0.240 to assign potential taxonomic identities. Data include all 3311 OTUs identified in our pipeline, before any were dropped due to rarefying. Available at https://doi.org/10.6084/m9.figshare.7500422.

File name: Supplementary Data 4
Description: **Phylogenetic tree of OTUs from this study and the Living Tree Project.** A phylogenetic tree in Newick format with tips for the 3,311 operational taxonomic units (OTUs) identified in the infant gut microbiome samples from this study identified using a USEARCH pipeline, and the 13,900 OTUs from the 132 release of the Living Tree Project. The topology of the tree reflects percent sequence similarity among taxa in the 16S rRNA V4 region (refer to Methods). Available at https://doi.org/10.6084/m9.figshare.7500563.

File name: Supplementary Data 5
Description: **Trait data mined from literature and online data repositories.** Trait data derived from the sources listed in Table 1 with Latin binomials that matched either those from the SILVA-based taxonomic identifications of OTUs found in infant gut microbiome samples, or from type specimens in the 132 release of the Living Tree Project. When observations existed for the same taxon across more than one data source, means were used. Available at https://doi.org/10.6084/m9.figshare.7501001.

File name: Supplementary Data 6
Description: **Trait data used in our analyses.** Data included trait values that could be directly associated to taxa in our study based on matching Latin binomials, and trait value estimates based on hidden state prediction using weighted square-change parsimony (refer to Methods and Supplementary Figure 8). Because we converted all trait values to numeric (e.g., Gram-negative = 0 and Gram-positive = 1), state predictions for initially discrete traits were allowed to be fractional (e.g., a Gram-positive score of 0.5), reflecting their probabilistic uncertainty. Available at https://doi.org/10.6084/m9.figshare.7501121.
